# Assessing risk profiles for *Salmonella* serotypes in breeding pig operations in Portugal using a Bayesian hierarchical model

**DOI:** 10.1186/1746-6148-8-226

**Published:** 2012-11-21

**Authors:** Carla Correia-Gomes, Theodoros Economou, Denisa Mendonça, Madalena Vieira-Pinto, João Niza-Ribeiro

**Affiliations:** 1 Instituto de Ciências Biomédicas Abel Salazar – Universidade do Porto (ICBAS-UP), Population Studies Department, Largo Prof. Abel Salazar, Porto, 2, 4099-003, PORTUGAL; 2 Instituto de Saúde Pública da Universidade do Porto (ISPUP), Rua das Taipas, Porto, 135, 4050-600, PORTUGAL; 3 CEMS, University of Exeter, Harrison Building, North Park Road, Exeter, EX4 4QF, UK; 4 UTAD, Veterinary Science Department, Apartado, Vila Real, 1013, 5001-801, PORTUGAL

## Abstract

**Background:**

The EU Regulation No 2160/2003 imposes a reduction in the prevalence of *Salmonella* in pigs. The efficiency of control programmes for *Salmonella* in pigs, reported among the EU Member States, varies and definitive eradication seems very difficult. Control measures currently recommended for *Salmonella* are not serotype-specific. Is it possible that the risk factors for different *Salmonella* serotypes are different? The aim of this study was to investigate potential risk factors for two groups of *Salmonella* sp serotypes using pen faecal samples from breeding pig holdings representative of the Portuguese pig sector.

**Methods:**

The data used come from the Baseline Survey for the Prevalence of *Salmonella* in breeding pigs in Portugal. A total of 1670 pen faecal samples from 167 herds were tested, and 170 samples were positive for *Salmonella.* The presence of *Salmonella* in each sample (outcome variable) was classified in three categories: i) no *Salmonella*, ii) *Salmonella* Typhimurium or *S*. Typhimurium-like strains with the antigenic formula: 1,4,5,12:i:-, , and iii) other serotypes. Along with the sample collection, a questionnaire concerning herd management and potential risk factors was utilised. The data have a “natural” hierarchical structure so a categorical multilevel analysis of the dataset was carried out using a Bayesian hierarchical model. The model was estimated using Markov Chain Monte Carlo methods, implemented in the software WinBUGS.

**Results:**

The significant associations found (when compared to category “no *Salmonella”*), for category “serotype Typhimurium or *S*. Typhimurium-like strains with the antigenic formula: 1,4,5,12:i:-” were: age of breeding sows, size of the herd, number of pigs/pen and source of semen. For the category “other serotypes” the significant associations found were: control of rodents, region of the country, source of semen, breeding sector room and source of feed.

**Conclusions:**

The risk factors significantly associated with *Salmonella* shedding from the category “serotype Typhimurium or serotype 1,4,5,12:i:-“ were more related to animal factors, whereas those associated with “other serotypes” were more related to environmental factors. Our findings suggest that different control measures could be used to control different *Salmonella* serotypes in breeding pigs.

## Background

*Salmonella* has been one of the major causes of food-borne disease in the European Union (EU) in the past years [1]. A considerable proportion of human cases are related to pork products [2]. The EU approved legislation (EU Regulation No 2160/2003) imposes a reduction on the prevalence of this agent in food production animals, such as pigs. To set the target for this reduction per country, baseline surveys were carried out in the EU to estimate the prevalence of *Salmonella* sp. in some food production animals. The objective of the surveys was to obtain comparable data for all Member States (MS) through a harmonized approach. These studies showed that the prevalence of *Salmonella* in holdings with breeding pigs was 31.8% (28.7% for breeding holdings and 33.3% for production holdings) [3] and also that there are different profiles in terms of serotype prevalence among different countries. In Portugal for instance, 9.1% of the breeding holdings were positive to *Salmonella* Typhimurium and 33.3% were positive to other serotypes than Typhimurium and Derby, while in Ireland these numbers were 17.5% for both cases [3]. Another important issue is that control programmes already being carried out in several MS have different efficiencies and so far, none seems to be able to reduce the level of *Salmonella* sp. to reach an eradication stage [4]. Control programmes should target all serotypes of *Salmonella* sp., since all of them have the potential to be pathogenic for humans. To improve the efficiency of control programmes, potential differences in serotypes prevalence which allows for differences in risk factors between serotypes should be taken in consideration. Some of the known risk factors in the literature are linked to: 1) biosecurity measures [5] especially those aimed at potential biological vectors (rodents) [6-8], hand, equipment and facility hygiene [9] and also purchase of animals from different suppliers [9]; 2) herd management - such as herd size [10], batch production system [11], housing - type of floor (partial slatted floor) [12,13] and type of pen [9]; 3) feeding practices such as dry feed [14], source of feed [15] and adding organic acids to feed [11]; 4) health disorders such as use of antibiotics [16,17], parasite infestations [18,19], and health status of the herd [11] among others. However, none of the aforementioned studies have taken into consideration whether risk factors differ between serotypes. To the best of our knowledge only one study compared the differences between risk factors for *Salmonella* serotypes with or without antimicrobial resistance [20]. The data for this paper were collected by the Portuguese Veterinary Authority (PVA) when the Baseline Survey on the Prevalence of *Salmonella* in breeding pigs was conducted in Portugal. The aim was to search for potential risk factors for shedding from two different groups of *Salmonella* serotypes using pen faecal samples from herds with breeding pig representative of Portuguese reality. The two groups were *Salmonella* Typhimurium including *S*. Typhimurium-like strains with the antigenic formula: 1,4,5,12:i:-, and other serotypes.

## Methods

### Herd selection

The objectives, the sampling frame, the diagnostic testing methods as well as the collection and reporting of data, and the timelines of the Baseline Survey on the Prevalence of *Salmonella* in breeding pigs were specified in the Commission Decision 2008/55/EC. The target population are holdings that constitute at least 80% of the breeding pig population in the Member State.

The sample size was calculated by the PVA and considered the number of swine herds existing in April of 2007, stratified by Region. The sampling frame consisted of 4522 herds, with 204,584 breeding pigs and 1,827,533 pigs in total. The herd inclusion criteria for entering the sampling frame were: to have at least 50 breeding pigs, either for breeding or production purposes. The pig population included in the sampling frame represented 87% of the total registered pig population in Portugal in 2007.The sample size was calculated using the sampling criteria specified in the Commission Decision 2008/55/EC Annex I - expected herd prevalence of 50%, desired confidence level of 95%, accuracy of 7.5% and then apply a finite population correction factor, with an increase of 10% for each group (breeding and production holdings) in case of non-response. A sample of 174 swine herds was randomly selected using probability proportional to the number of herds among the regions in Portugal.

### Pen selection

In each herd only the pens with breeding pigs over six months of age were randomly selected. The breeding pigs that have been recently introduced into the herd and were in quarantine were not included in the survey. In each selected herd, faecal samples from 10 pens were taken representing a 95% probability of detecting at least one positive sample if the true prevalence of infected pigs in the population was 10% [21]. The number of pens sampled per breeding room in each herd was allocated proportionally according to the number of breeding pigs in the different stages of production. The age categories in the sampling were not predetermined. The specification was that at least 10 individual breeding pigs should be included in each pooled pen faecal sample otherwise no sample was collected.

### Faecal samples collection

The faecal samples were collected and pooled together by the herd veterinary assistant and then sent to laboratory for detection of *Salmonella.* The material consisted of freshly voided faeces. Each pooled sample should weigh at least 25g and two approaches were employed to collect these pooled faeces samples: 1) where there was an accumulation of mixed faeces within an area of a pen or yard, a large swab was used to pass through the faecal mass, ensuring that at least 25g of mixed material was collected; 2) where there was no such accumulation (e.g. field, large yard, farrowing house, pens or other accommodation with low numbers of pigs per group) then individual pinches were selected from individual fresh faecal masses or places with a minimum of 10 individuals contributing to the final volume of at least 25g. The sites from which these pinches were collected were distributed in a representative manner across the area concerned. In approach 1) at least 10 individual pigs contributed to each sample taken, otherwise approach 2) was applied (Commission Decision 2008/55/EC).

### *Salmonella* isolation

At the laboratory, the isolation of *Salmonella* was done using the method described by Annex D of ISO 6579. The *Salmonella* strains isolated in the positive pen faecal samples were serotyped in the National Reference Laboratory for *Salmonella* according to Kaulfmann-White scheme. The sensitivity of cultured pooled faecal samples according to the described method varied around 80% and the specificity is 100% [22,23].

### Data collection

A questionnaire was used to collect information about the herd management and potential risk factors for *Salmonella* sp. shedding. This was filled by the herd veterinary who also collected the faecal samples (both tasks were conducted the same day). The questionnaire was designed by the PVA following the guidelines of Commission Decision 2008/55/EC. To minimize the bias that could be introduced by having different people collecting the data, the following procedures were taken: the majority of the questions were closed, the questionnaire had clear filling instructions attached and clarification meetings were held between the PVA and the field veterinarians before the sample collection took place. All the variables in the questionnaire are shown in Tables 1 and 2.

**Table 1:** Herd variables assessed by the questionnaire and distribution of the pen samples by the categories of the outcome variable

	**Number of pen samples by the categories of the outcome variable**				**Number of pen samples by the categories of the outcome variable**		
	**1**	**2**	**3**		**1**	**2**	**3**
**HERD VARIABLES**							
*Type of system*				*Management of breeding sows*			
Open air	29	0	1	more than 90% external source	*835*	*17*	*68*
Intensive	1242	38	110	>90% home raised	189	10	21
Missing observations	229	8	13	10-90% home raised	476	19	35
*Type of herd*				*Control of rodents*			
Selection and Multiplication Unit	292	8	30	No	1254	42	114
Production Unit	1208	38	94	Yes	*246*	*4*	*10*
*Region of the herd*				*Source of semen*			
Alentejo	229	8	13	Insemination centre – IC	491	11	18
Centre	278	8	34	Own boar + IC	869	23	88
Lisbon and Tagus Valley	*914*	*27*	*59*	Boar from another herd	*79*	*9*	*12*
North	79	3	18	Missing observations	*61*	*3*	*6*
*Type of production*				*Source of replacement pigs*			
Farrow-to-weaners	*164*	*5*	*21*	Just own herd	585	14	41
Farrow-to-growers	250	7	13	Others sources	906	31	83
Farrow-to-finish	794	26	60	Missing observations	9	1	0
Missing observations	292	8	30	Number of finishers pigs/herd			
*Number of boars*				<100	*263*	*3*	*14*
<3	*715*	*22*	*33*	≥100	1221	42	107
≥3	*785*	*24*	*91*	Missing observations	16	1	3
*Number of sows*				*Control of birds*			
<170	759	16	55	No	1192	34	94
≥170	741	30	69	Yes	*308*	*12*	*30*
*Number of gilts*				*Use of foot bath*			
<22	713	20	47	No	1017	38	85
≥22	787	26	77	Yes	483	8	39
*Size of the herd (number of breeding pigs)*				*Clothes for exclusive use in the herd*			
<203	759	16	55	Yes	1423	46	121
≥203	741	30	69	No	77	0	3
*Management of breeding boars*				*Herd replacement policy*			
more than 90% external source	606	24	70	Good	434	7	29
without boars or >90% home raised	735	15	40	Bad	1066	39	95
				*Biosecurity measures*			
10-90% external source or home raised	159	7	14	Yes	828	30	72
				No	672	16	52

Legend: 1 (no *Salmonella*), 2 (serotype Typhimurium or serotype 1,4,5,12:i:-), 3 (other serotypes).

**Table 2:** Pen variables assessed by the questionnaire and distribution of the pen samples by the categories of the outcome variable

	**Number of pen samples by the categories of the outcome variable**				**Number of pen samples by the categories of the outcome variable**		
	**1**	**2**	**3**		**1**	**2**	**3**
**PEN VARIABLES**							
*The pen has direct access to outside*				*Sanitary gap before breeders entering*			
No	1146	30	92	No	*626*	*19*	*44*
Yes	354	16	32	Yes	874	27	80
*Individual pen*				*Feed*			
No	1194	41	98	Dry pellet	229	7	27
Yes	306	5	24	Dry non pellet	*1230*	*36*	*97*
Missing observations	*0*	*0*	*2*	Wet	41	3	0
*Diarrhoea in the last 3 months*				*Floor*			
No	1445	45	118	Fully slatted	139	5	10
Yes	*33*	*1*	*2*	Others	1361	41	114
Missing observations	22	0	4	*Source of feed*			
*Age of the breeding sows*				Exclusively own	199	8	8
Only gilts or gilts and others	874	38	73	Bought + Mixture	*1301*	*38*	*116*
Without gilts	626	8	51	*Potential Salmonella control substances added to water*			
*Sex of the breeding pigs*				No	1291	38	111
Only females	1430	44	114	Yes	*209*	*8*	*13*
Males and females	*70*	*2*	*10*	*Use of antibiotics in the last 4 weeks in breeders*			
*Breeding sector*				No	1229	45	103
Mating room	210	10	21	Yes	*271*	*1*	*21*
Gestation room	789	26	62	*Way how was collected the sample*			
Mixture of room	58	1	14	Compose sample	121	1	11
Farrowing room	*390*	*7*	*22*	Swab	*1379*	*45*	*113*
Replacement breeders	*44*	*2*	*5*	Number of pigs in the pen			
				=10	1284	34	94
				>10	216	12	30

Legend:1 (no *Salmonella*), 2 (serotype Typhimurium or serotype 1,4,5,12:i:-), 3 (other serotypes).

### Data analysis

From the information gathered in the questionnaires, two new binary variables were created. The first variable groups the questions regarding management of replacing breeding pigs and their source, and was codified as Good if more than 90% of the breeding sows and boars were homebred (also included herds with no boars) and if the semen was not from another herd otherwise it was codified as Bad. The second variable combines the questions about biosecurity measures and was codified as Yes when controls for rodents and birds were implemented, and also if herds had provisions for foot bathing and clothe changing before entering the herd and No otherwise. The variables and their categories were recoded or aggregated to fewer categories as necessary to avoid sparse data problems as shown in Tables 1 and 2.

The continuous variables were transformed into categorical using the median values as the cut-off points defining the categories. Their summary statistics are shown in Table 3.

**Table 3:** Distribution of the continuous variables (at herd and pen level)

**Variable**	**Mean**	**Standard deviation**	**Minimum**	**Percentile 25**	**Median**	**Percentile 75**	**Maximum**
Number of boars	3.9	3.9	0	2	3	4	28
Number of sows	226.5	192.9	8	98	170	300	1077
Number of gilts	34.0	38.3	0	12	22	40	300
Size of the herd (number of breeding pigs)	265.0	216.9	41	109	203	355	1214
Number of pigs per pen*	11.6	8.0	10	10	10	10	130

* 258 pens had more that 10 pigs per pen.

Because of the low number of cases per serotype (Table 4), individual analysis of each *Salmonella* serotype was prohibitive. Therefore the outcome variable was the isolation of *Salmonella* in each sample and was classified in three categories: i) no *Salmonella*, ii) serotype Typhimurium and *S.* Typhimurium-like strains with the antigenic formula: 1,4,5,12:i:-, and iii) other serotypes. For the calculation of apparent herd prevalence, a herd was considered positive if it had at least one positive pen faecal sample. The percentage of positive *Salmonella sp.* pen faecal samples was 27% *Salmonella* Typhimurium or *S.* Typhimurium-like strains with the antigenic formula: 1,4,5,12:i:-, and 73% other serotypes.

**Table 4:** Percentage of serotypes isolated in the study

**Serotype**	**Percentage of isolates (n)**			**Percentage of herds that have at least one pen sample positive to the serotype (n)**		
	**Breeding holdings**	**Production holding**	**All Holdings**	**Breeding holdings**	**Production holding**	**All holdings**
Typhimurium	15.8 (6)	25 (33)	23 (39)	13.6 (3)	25.6 (20)	13.2 (23)
Rissen	18.4 (7)	19.7 (26)	19 (35)	22.7 (5)	19.2 (15)	12.0 (20)
London	21 (8)	13.6 (18)	15 (26)	13.6 (3)	11.5 (9)	7.2 (12)
Derby	15.8 (6)	9.1 (12)	11 (18)	13.6 (3)	8.9 (7)	6.0 (10)
Give	13.1 (5)	5.3 (7)	7 (12)	9.1(2)	5.1 (4)	4.0 (6)
Brandenburg	0 (0)	6.1 (8)	5 (8)	0 (0)	2.6 (2)	1.8 (2)
1,3,19:-:-	2.6 (1)	4.5 (6)	4 (7)	4.5 (1)	6.4 (5)	3.6 (6)
1,4,5,12:i:-	5.3 (2)	3.8 (5)	4 (7)	9.1 (2)	3.8 (3)	3.0 (5)
Bovismorbificans	0 (0)	3 (4)	2 (4)	0 (0)	2.6 (2)	1.2 (2)
Gloucester	0 (0)	2.3 (3)	2 (3)	0 (0)	2.6 (2)	1.2 (2)
Muenchen	2.6 (1)	2.3 (3)	2 (4)	4.5 (1)	3.8 (3)	2.4 (4)
Anatum	0 (0)	1.5 (2)	1 (2)	0 (0)	2.6 (2)	1.2 (2)
Bredeney	0 (0)	0.8 (1)	1 (1)	0 (0)	1.3 (1)	0.6 (1)
Goldcoast	0 (0)	1.5 (2)	1 (2)	0 (0)	1.3 (1)	0.6 (1)
Livingstone	2.6 (1)	0 (0)	1 (1)	4.5 (1)	0 (0)	0.6 (1)
Mbandaka	2.6 (1)	0.8 (1)	1 (2)	4.5 (1)	1.3 (1)	1.2 (2)
Senftenberg	0 (0)	0.8 (1)	1 (1)	0 (0)	1.3 (1)	0.6 (1)

The data have a “natural” multilevel structure: pen faecal samples (first level) nested in herds (second level) and were analysed using a Bayesian hierarchical model with a categorical response variable (three categories). Monte Carlo Markov Chain (MCMC) was used for estimation and this was implemented in the freely available software WinBUGS (BUGS project, http://www.mrc-bsu.cam.ac.uk/bugs/winbugs/). Hierarchical models are naturally handled in the Bayesian framework because of the conditional independence assumed between each level in the hierarchy. In conjunction with the open-source software WinBUGS, this provides a general framework for implementing hierarchical models in similar applications.

Random effects were included at the herd level to account for the fact that the observations are ‘nested’ in herds. Treating the herd effect as random, also allows for the fact that the number of herds here (167) is a sample of all existing herds. All prior distributions were chosen to be as uninformative as possible. A more detailed description of the model is given in Additional file 1.

To decide which variables should be included in this multivariable model, an exploratory analysis was performed by fitting univariable models and considering as candidates for the multivariable model, all variables significant at the 0.15 significance level. Associations between the explanatory variables were tested using a chi-square test and if a significant association (p < 0.05) was found, only the variables with more biological justification were allowed to enter the model.

The final multivariable model was built using a forward selection process until all variables with a significant 95% credible interval were included. The significance level was set at 0.05.

The model ran long enough with sufficient burn-in (5000 iterations) to ensure convergence to the posterior distribution of the parameters. Convergence was assessed by visual inspection of the means in time-series plots but also more formally using the Raftery and Lewis, and the Gelman-Rubin R-hat diagnostics [24,25]. R-hat should be arbitrarily close to 1 for convergence. The chains were thinned by only collecting 1 in 10 consecutive samples and this eliminated autocorrelation in posterior samples (using the CODA package [26] in R). Mixing in the chains was assessed by comparing the MC (Markov Chain) error with the standard deviation, for each parameter. Ideally, the MC error should be less than 5% of the standard deviations for good mixing [27] and this was true for all parameters here. Two MCMC chains ran with dispersed initial values which is good practice to ensure convergence and mixing. WinBUGS code for implementing the model is given in Additional file 2.

The presence of confounding was investigated by analysing the correlation matrix of the joint posterior distribution for all model parameters but especially the slope parameters. Correlation values higher than 0.5 where takes to indicate significant correlation.

Posterior predictive simulation was used for model checking as described by Gilks et al. [28]. This technique is effectively testing whether the observed data are extreme in relation to the predictive distribution (fitted model). Model deviance was the measure adopted for comparison. The technique involves the estimation of a p-value which should not be extreme (close to 0 or 1) for good model fit.

## Results

A total 167 herds (33 breeding and 134 production holdings) responded to the questionnaire and were tested: 76 herds were positive to *Salmonella* sp. (apparent prevalence of 45.5%, CI: 37.9% - 53.1%). Of these, 15 breeding holdings (apparent prevalence of 45.5%, CI: 28.5% - 62.4%), and 61 production holdings (apparent prevalence of 45.5%, CI: 37.1% - 53.9%) were positive to *Salmonella* sp. Among the 1670 faecal samples collected, 170 were positive (10.1%) and seventeen different serotypes were found (Table 4). There was no simultaneous occurrence of the two groups of serotypes in any of the positive samples. *Salmonella* Typhimurium was found in 23% of the positive isolates (15.8% in breeding and 25% in production holdings), followed by *Salmonella* Rissen (19%) (Table 4). The proportion of the different serotypes by type of holding is detailed in Table 4. Considering the distribution of serotypes groups through the herds, it was observed that 13.8% of the herds had at least one sample positive to serotype Typhimurium or *S*. Typhimurium-like strains with the antigenic formula: 1,4,5,12:i:-, and 31.7% of the herds had at least one positive sample to other serotypes. A significant association was found between number of sows and number of breeding pigs and for this reason it was decided that only the number of breeding pigs should enter the multivariable model.

Several management practices linked to herd and pen were assessed (Tables 1 and 2). The variables - region of the herd, size of the herd, source of semen, rodents control, number of pigs per pen, age of breeding sows, breeding sector room, source of feed and use of antibiotics - were selected to enter the multivariable model. Table 5 shows the final multivariable multilevel model results. The results were converted to odds ratio (OR) and the respective 95% credible intervals (OR CrI) were calculated. The posterior median was used to estimate point values of each OR, because unlike the mean, this is less affected by asymmetric distributions. Posterior distributions of all OR are highly asymmetric since they are based on the exponentiation of posteriors of the slope parameters. The convergence of MCMC calculations was considered acceptable with R-hat values of all parameters being less than 1.001. Different starting values did not affect the final results. None of the between-parameter correlations was larger than 0.5 in magnitude while the majority was less than 0.1 implying no influential confounding in any of the variables. Significance of the herd random effects is tested by looking at whether the variance estimates (1/τ_1_, 1/τ_2_) are non-zero. Estimates of 1/τ_1_ and 1/τ_2_ are arbitrarily away from zero (5.8 and 1.4 respectively) and their standard errors are relatively small, indicating that both estimates are different from zero (see Table 5). The model fit was reasonably accurate with a p-value of 0.21 which means no significant differences between replicated and observed data.

**Table 5:** **Posterior results for the final multivariable categorical multilevel model for the risk factors (*Salmonella *negative as reference group)**

**Variable**	**Typhimurium or 1,4,5,12:i:-**				**Other serotypes**			
	**Coefficient**	**SD**	**OR**	**95% OR CrI**	**Coefficient**	**SD**	**OR**	**95% OR CrI**
HERD								
Region of the herd								
Alentejo	0		1.0		0		1.0	
Centre	−1.3	1.5	0.28	0.01-4.30	1.5	0.7	**4.57**	**1.33-17.57**
Lisbon and Tagus Valley	−0.5	1.1	0.62	0.07-5.05	0.9	0.6	2.56	0.86-8.36
North	−0.1	1.7	0.88	0.03-24.31	2.6	0.8	**12.9**	**2.97-64.33**
Size of the herd: (number of breeding pigs)								
<203	0		1.0		0		1.0	
≥203	1.9	0.9	**7.04**	**1.46-60.04**	0.5	0.4	1.65	0.83-3.44
Source of semen								
Insemination centre – IC	0		1.0		0		1.0	
Own boar + IC	0.4	0.8	1.45	0.24-7.77	1.1	0.4	2.91	1.35-6.83
Boar from another herd	3.7	1.6	**41.22**	**2.46-1392.7**	1.4	0.8	4.18	0.94-19.30
Control of rodents								
No	0		1.0		0		1.0	
Yes	−2.2	1.8	0.11	0.002- 1.85	−2.0	0.7	**0.13**	**0.03-0.45**
PEN								
Number of pigs/pen								
=10	0		1.0		0		1.0	
>10	1.4	0.7	**4.06**	**1.03-19.73**	0.6	0.4	1.82	0.88-3.79
Age of the breeding sows								
Only gilts or gilts and others	0		1.0		0		1.0	
Without gilts	−1.8	0.8	**0.17**	**0.03-0.65**	0.2	0.3	1.24	0.68-2.24
Breeding sector room								
Mating	0		1.0		0		1.0	
Gestation	0.1	0.5	1.11	0.44-3.10	−0.2	0.3	0.81	0.45-1.52
Mixture of animals of different sectors	0.2	1.7	1.17	0.03-24.80	0.8	0.7	2.14	0.54-7.78
Farrowing	−1.0	0.6	0.36	0.10-1.22	−1.0	0.4	**0.38**	**0.17-0.80**
Replacement breeders	−0.9	1.1	0.40	0.04-2.72	0.1	0.7	1.15	0.29-3.88
Source of feed								
Exclusively own	0		1.0		0		1.0	
Not exclusively own	0.5	1.1	1.63	0.18-17.62	2.0	0.7	**7.29**	**2.25-29.46**
Herd random effect variance	5.8	0.66			1.4	0.24		

Legend: SD – standard deviation, OR – odds ratio, CrI – credible interval, in bold the significant OR for a 95%CrI.

It can be seen from the analysis of Table 5 that there are different risk profiles for the two *Salmonella* serotype categories when compared to category “no *Salmonella”*. This is an important finding and suggests that the risk factors may be different between the categories of serotypes defined in this study. For category “Typhimurium or *S*. Typhimurium-like strains with the antigenic formula: 1,4,5,12:i:- associations with significant change in risk were: 1) *size of the herd*: herds with 203 and more breeding pigs are at higher risk of infection, 2) the source of semen: purchase of boars from other herds increase the risk of infection, 3) *number of pigs per pen*: pens with more than 10 animals per pen have increased risk of infection, and 4) the age of the sows: pens without gilts have a decreased risk of infection. For the category “other serotypes” the significant risk associations were: 1) *region of the herd*: herds in the Centre and North Region have a higher risk of infection, 2) *the source of semen*: the use of own boar increased the herds’ risk of infection, 3) *control of rodents* had a significant effect in reducing the risk at the herd level, 4) *feed source:* using feed from external sources, i.e., not exclusively from the farm increased the risk of infection, and 5) *breeding sector*: the farrowing sector had a lower risk of infection than the mating sector.

## Discussion

This study investigated risk factors for *Salmonella* shedding for two different groups of serotypes using pen faecal samples from herds with breeding pigs adequately representing the Portuguese pig industry.

### The outcome variable

The different serotypes of *Salmonella sp.* were divided in two groups because serotype Typhimurium is a serotype with a recognized difficult control [29] and is also the cause of many human cases of food-borne disease linked to pork meat. Serotype Typhimurium-like strains with the antigenic formula: 1,4,5,12:i:-was included in the group of serotype Typhimurium because of the genetic similarity, the similar virulence and the antimicrobial resistance characteristics existing between the two serotypes [30]. The use of composite samples increases the overall sensitivity of detection of infected pens [31] strengthening the confidence on the accuracy of our response variable. The increased sensitivity of the use of pooled faecal samples was shown by the analysis of the Baseline Survey results [32] which demonstrated that this pooled sampling process detected approximately 80% of the true *Salmonella* positive herds, and that with 10 pooled faecal samples it is possible to detect at least one positive sample in a pig herd when the animal level prevalence is at least 20%, with 95% certainty [31].

### The model

It was anticipated that the hierarchical structure of the data from our sample could influence the outcome of the analysis. Therefore the statistical approach was chosen to take into consideration the multilevel structure of data from our sample where the pen faecal samples (level 1) are nested in herds (level 2). Some important remarks concerning the statistical approach deserve to be highlighted: the model implemented here showed a good fit, despite the fact there was little information to update the prior distributions. The methodology proposed could offer a general modelling approach to researchers who want to incorporate expert knowledge in the specification of the priors or for those who wish to restrict the priors accordingly to account for lack of information in the response variable which was not the case in this study. Lastly, both WinBUGS and R, are freely available software which is particularly appealing for the purpose of presenting the methodology here as a general modelling tool.

### Risk factors for *Salmonella* Typhimurium and Typhimurium-like strains with the antigenic formula: 1,4,5,12:i:- infection

It can be seen from the analysis of Table 5 that there are different risk profiles for the two categories of *Salmonella*, validating our initial hypothesis that the risk factors could vary between the two categories of serotypes studied.

In category “Typhimurium or 1,4,5,12:i:-” *the size of the herd* (the number of breeding pigs being equal or greater than 203) was considered a risk factor. A similar association was found for *Salmonella* sp. in finishers [10] and also in the breeding pigs [32]. A reason for this is that in bigger herds, the risk of transmission is higher given a higher number of “infectious” and “susceptible” animals, offering increased chances of more effective contacts per unit of time. The *number of pigs per pen* was another risk factor, already reported for *Salmonella sp.* in breeding pigs by Nollet in 2005 [32]. As in the case of the size of the herd, the greater the number of pigs in the pen, the easier the transmission of infection between pigs, if there are infected pigs in that pen. Interestingly, these two factors were not found significant for “other serotypes” which suggests that “serotype Typhimurium category” could be more associated with transmission between animals than other categories. A protective association, relating to *pens without gilts* was found. A similar association was also found in the European Union Baseline survey on breeding pigs for maiden gilts [32]. One reason may be that older sows have higher immunity status to *Salmonella* Typhimurium and may be less susceptible to stress than younger sows although they could be carriers (the test used was pooled faecal culture so it could not detect carriers if they are not shedding) [33]. The last significant risk factor found in this category of the outcome was the *boar from another herd* which however, has a wide credible interval. A combination of the high odds ratio with a relatively small number of pen faecal samples in this variable category indicates that this association should be a matter of further study. Interestingly, for *rodent control*, a strong protective effect was noticed towards the Typhimurim group (noticeable by the OR = 0.11) although not statistically significant. However, it is our opinion that rodent control should not be disregarded from the list of risk factors for *S.* Typhimurim.

### Risk factors for infection with other serotypes of *Salmonella*

Concerning the category “other serotypes”, the *region of the herd* was found relevant: samples from herds in the North and Centre Region had higher odds of being positive than samples from herds in the Alentejo Region. Possibilities to explain this finding are that herds in the Centre and North regions are close together or share common management factors. This variable needs further studying to understand whether there are differences in management procedures that were not evaluated by this questionnaire, as this variable did not influence the results of the other variables when it entered the model. Using *semen from own boar* is a risk factor when compared to using semen from insemination centres only, where the animals are tested and if *Salmonella*-positive culled. This association has not been reported yet in the literature, probably because in the majority of the countries the semen comes from insemination centres. Pens where pigs *feed is not exclusively home produced* were at higher risk: the risk is linked to exotic serotypes such as the ones that are isolated in commercial feed; similar association was also found in other studies but for *Salmonella* sp. [15,32]. There is a protective effect for farrowing pens when compared to mating pens. This can be justified by the hormonal changes in the sow at mating which is similar to the results found in a longitudinal study for sows seven days after weaning [33] where more *Salmonella* was detected at mating than in the others sector of breeding sows. In that study, this was attributed to hormonal changes that takes place in the sows resulting in follicular growth, ovulation and oestrus behaviour, and also to rise in adrenocorticotrope hormone due to stress. So it was concluded that with stress sows are more susceptible to infection and also carrier sows are more likely to start shedding the pathogen [33]. The control of rodents was considered a protective factor for the presence of “other serotypes”: the role of rodents in the transmission of this agent was also highlighted in other studies [6,8]. Since rodents could lead to the dissemination of the agent in the herd as a vector that transmits the infection between closed sectors their role must not be underestimated in a control programme. As already mentioned this variable appears as a protective factor to the group Typhimurium or 1,4,5,12:i:- although not statistically significant. This intriguing finding does not compromise the hypothesis of the importance from pig to pig transmission – direct or indirect - in the case of Typhimurium.

### Application in control

The results from this work should be taken into account when implementing control and biosecurity programmes to *Salmonella* sp., since they highlight the importance to pre-define herd infection status regarding *S.* Typhimurium, and of making a risk profile based on the management practices in place before the adoption of control measures. Control measures should be adapted to suite the type of infection present bearing in mind that for serotype Typhimurium the control of animal source risk factors should be considered, whereas for the other serotypes is it the environmental source risk control that is important.

## Conclusion

In Portugal, the prevalence of herds with breeding pigs that had at least one sample positive to serotype Typhimurium or *S*. Typhimurium-like strains with the antigenic formula: 1,4,5,12:i: was 13.8% and for the other serotypes 31.7%. A flexible and innovative statistical modelling approach was successfully used here. This provides a framework for similar studies of other diseases as it is straightforward to implement and can be easily generalized. The risk factors for serotype Typhimurium suggest a contagious pattern and the risk factors for other serotypes appeal to be related to environmental factors. The role of rodent control in serotype Typhimurium needs further studies. This study provided valuable information that can be incorporated in future control programmes for *Salmonella* sp. in breeding pigs in Portugal and other countries.

## Competing interests

The authors do not have any competing interest.

## Authors’ contributions

CCG was involved in the design and performed the statistical modelling analysis and drafted the manuscript. TE performed part of the statistical analysis and was involved in the revision of the manuscript for intellectual contents. DM was involved in the revision of the manuscript for intellectual contents and statistical analysis. MVP was involved in the revision of the manuscript for intellectual contents. JNR was involved in the design of the statistical analysis, in the drafting and revision of the manuscript for intellectual content. All authors approved the final manuscript.

## Supplementary Material

Additional file 1 Model framework. Click here for file

Additional file 2 WinBUGS code for the categorical multilevel model. Click here for file
