# Supplementary material for: Assessing risk profiles for Salmonella serotypes in breeding pig operations in Portugal using a Bayesian hierarchical model
Source: BMC Vet Res. 2012 Nov 21;8:226. doi: 10.1186/1746-6148-8-226 (PMC3514327; doi:10.1186/1746-6148-8-226)
Supplement: Additional file 1 — Model framework. [file 1746-6148-8-226-S1.docx]

**Additional File 1 – Model framework**

Level 1- pen samples:


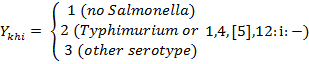


where

$\begin{matrix} Pr\left(Y_{khi}\right)=p_{khi} ;k=1,2,3 for outcome variable;h=1,\ldots1670 for samples ; i=1,\ldots,167 for herds and \\ \sum_{k=1}^{3} \text{p}_{khi}=1 \end{matrix}$is the probability of occurrence for each category of the outcome variable Y. These probabilities are themselves modelled using explanatory variables and random effects:

$$\begin{matrix} logit\left(p_{khi}\right)= \alpha_{k}+ \beta_{jk}\text{herd variables}_{ih}+\beta_{jk}\text{pen variables}_{h}+\beta_{jk}\text{herd variables}_{ih}\text{*herd variables}_{ih} \\ \begin{matrix} & & \end{matrix}+\beta_{jk}\text{pen variables}_{h}\text{*pen variables}_{h}+\beta_{jk}\text{herd variables}_{ih}\text{*pen variables}_{h}+b2_{ik} \text{ } \end{matrix}$$

where j is the number of explanatory variables.

Note that with the use of random effects, the probabilities of Y=1, 2 or 3 are herd specific.

The probability for each category of Y is modelled using the same explanatory variables but different slope parameters (β_jk_) to assess whether those variables affect each category in a different way. The reference category is Y=1 (no *Salmonella*) and all the results from each of the categories Y=2 and 3 are compared to the reference category.

Level 2 - herds:

$$b2_{i1}\begin{matrix} = & 0 \end{matrix}$$

$$b2_{i2}\sim N(0,1/\tau_{1})$$

$b2_{i3}\sim N(0,1/\tau_{2})$ where 1/τ_1_ and 1/τ_2_ are the variances for category “serotype Typhimurium or serotype 1,4,5,12:i:-” and “other serotypes” respectively.

The b2_ik_ are the random effects allowing for the fact that the observations are 'nested' in herds (this reduces the effective number of model parameters by ‘pooling’ herd information, while retaining model flexibility). Treating the herd effect as random, also allows for the fact that the number of herds here (167) is a sample of all existing herds.

The prior distributions for the model parameters:


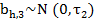

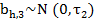

$$\alpha_{1}=0$$

$\alpha_{k}\sim N(0,100)$ where k = 2,3 for the intercepts in each category of Y_khi_.

$\beta_{j1}=0$ where j = 1,2,…,14 for the reference category of the explanatory variables.


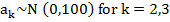

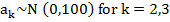

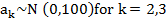

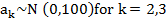

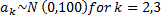

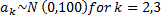

$\beta_{jk}\sim N(0,100)$ where j = 1,2,…,14 and k =2,3. These are the fixed effects of the explanatory variables in the other two categories of the Y_khi_.


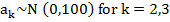

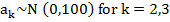

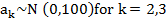

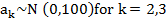

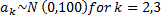

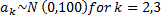

$\tau_{1}\sim Gamma(0.5,0.001)$,

$\tau_{2}\sim Gamma(0.5,0.001)$ for the variance of the herd random effects.

All prior distributions were chosen to be as uninformative as possible. For parameters with infinite support, Gaussian priors with large variance are conventionally used to express lack of information [[34](#_ENREF_34)]. For variance parameters with strictly positive support, the inverse of the variance (precision) is given an uninformative gamma distribution implying that the variance is given an inverse gamma. The inverse gamma is the conjugate prior for a Gaussian random effect therefore it is a natural choice which aids computation. A Gamma(0.5,0.001) was chosen which has mean 500 and variance of 500000, implying it is a very flat or uninformative prior distribution.
