# Supplementary material for: Assessing risk profiles for Salmonella serotypes in breeding pig operations in Portugal using a Bayesian hierarchical model
Source: BMC Vet Res. 2012 Nov 21;8:226. doi: 10.1186/1746-6148-8-226 (PMC3514327; doi:10.1186/1746-6148-8-226)
Supplement: Additional file 2 — WinBUGS code for the categorical multilevel model. [file 1746-6148-8-226-S2.docx]

**Additional File 2 – WinBUGS code**

Figure1. WinBUGS code for the categorical multilevel model

Legend: H = number of cases, K = number of categories in the outcome variable = 3 (1-no *Salmonella*, 2 - serotype Typhimurium or serotype 4,5,12:i-, 3 - other serotypes), sero[h] = outcome variable, cod.herd[h] = number of the herd, α = intercept for each outcome result, β = fixed effects, b2 = random effects considering herd level, I = number of herds, Variables: rod = rodents control, sem2 = mixture of own boar semen and semen form insemination centres, sem3 = semen of boar from another herd, reg2 = Centre region, reg3 = Lisbon and Tagus Valley region, reg4 = North region, herdsize = size of the herd: number of breeding pigs (equal or more than 203), feed = source of feed in the pen, sec2 = gestation pen, sec3 = mixture of animals of different sector in the pen, sec4 = farrowing pen, sec5 = replacement breeders pen, num = more than 10 animals per pen, age = age of the breeding sows in the pen, sig1 = standard deviation of category Typhimurium or 4,5,12:i-, sig2 = standard deviation of category other serotypes
